# Polyamines in Halophytes

**DOI:** 10.3389/fpls.2019.00439

**Published:** 2019-04-09

**Authors:** Milagros Bueno, María-Pilar Cordovilla

**Affiliations:** Laboratory of Plant Physiology, Department of Animal Biology, Plant Biology and Ecology, Faculty of Experimental Sciences, University of Jaén, Jaén, Spain

**Keywords:** antioxidant system, extremophiles, ion sequestration, saline markers, wetlands species, xerohalophytes

## Abstract

Polyamines (PAs) are related to many aspects of the plant’s life cycle, including responses to biotic and abiotic stress. On the other hand, halophytic plants are useful models for studying salt tolerance mechanisms related to the adaptive strategies that these plants present in adverse environments. Furthermore, some halophytes have high economic value, being recommended instead of glycophytes as alternative agricultural crops in salt-affected coastal zones or saline farmlands. In recent years, the understanding of the role of PAs in salt-tolerant plants has greatly advanced. This mini review reports on the advances in the knowledge of PAs and their participation in achieving better salt tolerance in 10 halophytes. PAs are associated with responses to heavy metals in phytoremediation processes using certain salt-tolerant species (*Atriplex atacamensis*, *A. halimus*, *Inula chrithmoides*, and *Kosteletzkya pentacarpos*). In crops with exceptional nutritional properties such as *Chenopodium quinoa*, PAs may be useful markers of salt-tolerant genotypes. The signaling and protection mechanisms of PAs have been investigated in depth in the extreme halophyte *Mesembryanthemum crystallinum* and *Thellungiella* spp., enabling genetic manipulation of PA biosynthesis. In *Prosopis strombulifera*, different biochemical and physiological responses have been reported, depending on the type of salt (NaCl, Na_2_SO_4_). Increases in spermidine and spermine have been positively associated with stress tolerance as these compounds provide protection in *Cymodocea nodosa*, and *Solanum chilense*, respectively. In addition, abscisic acid and salicylic acid can improve the beneficial effect of PAs in these plants. Therefore, these results indicate the great potential of PAs and their contribution to stress tolerance.

## Introduction

Halophytes comprise a group of plants able to complete their life cycle under saline environmental conditions of around 200 mM NaCl or even more (Flowers and Colmer, 2008; Golldack et al., 2014). Representing roughly 1% of the total world flora, they are distributed mainly in arid and wetlands saline areas, as well as in temperate zones (Gul et al., 2013; Kumari et al., 2015). These plants provide useful models to understand the mechanisms of adaptation to saline stress (Hurst et al., 2004; Arbona et al., 2010). The salt-tolerance strategies include: accumulation or exclusion of ions, control and compartmentalization of ion uptake, maintenance of osmotic balance (Shabala and Mackay, 2011; Bose et al., 2014), biosynthesis of compatible solutes (Flowers and Colmer, 2015; Slama et al., 2015), shift in the photosynthetic pathway (Uzilday et al., 2015), activation and synthesis of antioxidant compounds (Ozgur et al., 2013), induction and modulation of plant hormones (Gupta and Huang, 2014; Fahad et al., 2015), and the modulation of polyamines (PAs) (Shi and Chan, 2014; Liu et al., 2015). The use of mutants, transgenic plants, or exogenous PAs has revealed the key role of these compounds in multiple responses to salt stress (Liu et al., 2014; Parvin et al., 2014; Pathak et al., 2014).

Polyamines are polybasic aliphatic amines, widely distributed in all living organisms and involved in multiple cellular functions (Kusano et al., 2008; Hussain et al., 2011; Gupta et al., 2013; Tiburcio et al., 2014). The most commonly occurring PAs, found in halophytes, are the diamines putrescine (Put) and cadaverine (Cad), the triamine spermidine (Spd), and the tetramine spermine (Spm) (Shevyakova et al., 2006b; Kuznetsov et al., 2007). These compounds may serve as bioindicators of stress-tolerant lines (Simon-Sarkadi et al., 2007), stabilize biological membranes, regulate ion homeostasis, delay senescence processes (Lutts et al., 2013), regulate membrane transport through a direct interaction with plasma membrane or vacuolar transporters (Pottosin and Shabala, 2014), protect photosynthetic tissues (Malliarakis et al., 2015), regulate the antioxidant system and free-radical machinery (Sudhakar et al., 2015), participate in abiotic stress signaling (Pál et al., 2015), and protect against metal toxicity (Ghabriche et al., 2017; Zhou et al., 2018a,b). Furthermore, PAs also activate genes for stress response and interact with other metabolic pathways by establishing hormonal cross-talk (Pál et al., 2015; Llanes et al., 2018). Most studies on PAs have been made in glycophytes. The present review attempts to consolidate our understanding of the effects of PAs and their role in the physiology of 10 halophytes (Table 1), helping to elucidate the potential mechanisms of these growth regulators in halophytic plants.

**Table 1:** List of revised halophytic species where Polyamines have been studied.

Scientific name	Polyamines found	Functions involved	References
*Atriplex atacamensis*	Putrescine Spermidine Spermine	Toxic ion sequestration	Vromman et al., 2011
*Atriplex halimus*	Diaminopropane Putrescine Spermidine Spermine	Salt excretion Decrease in heavy metal toxicity	Ben Hassine et al., 2009; Lefèvre et al., 2010
*Chenopodium quinoa*	Putrescine Spermidine Spermine	Maintaining ion balance Markers of salt tolerance	Orsini et al., 2011; Ruiz-Carrasco et al., 2011; Ruiz et al., 2017
*Cymodocea nodosa*	Putrescine Spermidine Spermine	Protective role on photosynthetic apparatus	Zarranz Elso et al., 2012
*Inula chrithmoides*	Cadaverine Putrescine Spermidine Spermine	Protection of endogenous cellular structures	Ghabriche et al., 2017
*Mesembryanthemum crystallinum*	Agmatine Cadaverine Diaminopropane Putrescine Spermidine Spermine	Mitigation of oxidative stress Photochemical efficiency in photosystem II Stress signaling	Shevyakova et al., 2006a,b, 2011, 2013; Kuznetsov et al., 2007; Stetsenko et al., 2009; Surówka et al., 2016
*Prosopis strombulifera*	Cadaverine Diaminopropane Putrescine Spermidine Spermine	Stimulation of growth and probably related to the antioxidant defense system	Reginato et al., 2012
*Solanum chilense*	Putrescine Spermidine Spermine	Protective functions	Gharbi et al., 2016
*Thellungiella spp.*	Putrescine Spermidine Spermine Cadaverine	Favoring osmoregulation Stabilizing proteins Antioxidant activity Protection from UV-B	Radyukina et al., 2007, 2017; Zhou et al., 2015; Ping Lee et al., 2016
*Kosteletzkya pentacarpos*	Putrescine Spermidine Spermine	Reducing the accumulation of heavy metals	Zhou et al., 2018a,b

## Halophytes as Model Plants to Study Polyamines

Shevyakova et al. (2006b) were the first to investigate the free PA content in *Mesembryanthemum crystallinum* L. (the common ice plant) a C_3_-CAM (crassulacean acid metabolism) plant (Adams et al., 1998; Winter and Holtum, 2007), under severe salinity conditions. The dynamics of Spm accumulation resembled that of phosphoenolpyruvate carboxylase (PEPC), a key enzyme of the water-saving CAM pathway; this could indicate an indirect involvement of PAs in plant adaptation to stress. The gradual increase in Spm could be due to several causes: the conversion of Spd into Spm, the release of free Spm (active) from its conjugates, changes in the biosynthetic rate, and the degradation and/or competition between PAs and ethylene based on alterations in sulfur metabolism, necessary for S-adenosyl methionine (SAM) synthesis (a precursor of both PAs and ethylene). Shevyakova et al. (2006a) and Stetsenko et al. (2009) showed the accumulation of free and conjugated forms of PAs, although the latter predominated under saline conditions. However, for Cad, conjugated forms underwent a transition into the free form. These results indicate considerable quantitative organ-specific changes in the pool of free and conjugated forms, regulated by the combined action of H_2_O_2_-peroxidase and the oxidative degradation of PAs. 1.3-diaminopropane (Dap) was located in roots and was related with protection of membranes. Kuznetsov et al. (2007) suggested that accumulation of Cad could compensate for the Put deficit, acting as a DNA protector and a free-radical scavenger. Shevyakova et al. (2011) also demonstrated an antioxidant role of PAs. Spd diminished the expression of ferritin gene and consequently the production of reactive oxygen species (ROS). Shevyakova et al. (2013) found a protective effect of ABA related to the regulation of proline (Pro), PAs, and cytokinin contents. Under saline conditions, ABA treatment increased free PA content, in roots, whereas that in leaves, could regulate conjugate forms, thus controlling the PA biological functions. ABA may also be related to the transport of Cad from roots (decreased levels) to leaves (increased levels). The mechanisms of signaling and protection of PAs in *M. crystallinum* were reported by Surówka et al. (2016). These researchers studied (under exogenous application of H_2_O_2_) the diurnal rhythm cycle of antioxidants and osmotic compounds (transition C_3_-CAM), which could be responsible for the maintenance of the water-potential gradient, ROS homeostasis, and the prevention of oxidative damage. Among these components, PAs (accumulation of Put and Spd but lower amounts of Spm and agmatine) showed a daily pattern of accumulation, similar to malate, suggesting a metabolic connection between the two routes. On the other hand, the accumulation of these amines could be related to mitigate oxidative stress, and maintenance maximum photosystem II (PS II) photochemical efficiency (*Fv/Fm*) (Kotakis et al., 2014). These mechanisms regulate cellular metabolism and trigger signaling cascades leading to stress acclimation.

Radyukina et al. (2007) studying a new model plant *Thellungiella halophila*, found a high capability of accumulation of Na^+^ and Cl^−^ ions, in their roots and leaves, under progressing salinity. In addition, they observed Pro accumulation due to enhanced expression of biosynthetic genes, accumulation of Spd and later Spm, increased expression of SAM synthetase and Spd synthase genes, and high constitutive levels of peroxidase that palliates the severity of oxidative stress. Later, Zhou et al. (2015) studied a novel Spd synthase gene (*EsSPDS1*), which was cloned and characterized from the halophytic plant *Eutrema salsugineum*. The expression of this gene was induced by polyethylene glycol (PEG), NaCl and ABA treatments. In transgenic tobacco plants, *EsSPDS1*-overexpressing lines exhibited drought tolerance via the reduction of water loss and the increase of sugar and Pro, favoring osmoregulation, stabilizing protein structure, and reducing oxidative potential. Overexpression of *EsSPDS1* lowered the levels of malondialdehyde (MDA), diminished ion leakage, and also activated the antioxidant enzymes superoxide dismutase (SOD) and catalase (CAT), which mitigated oxidative stress. These data suggest that the activation of the *EsSPDS1* gene could be an option for developing drought-tolerant plants. Ping Lee et al. (2016), working with diverse accessions of *Thellungiella* spp. collected from different geographical origins in many saline habitats, found that all accessions survived and grew with up to 700 mM NaCl. Salt stress induced the accumulation of high amounts of osmolytes. With respect to PAs, Put levels increased linearly with salt concentrations in all accessions. These researchers indicated a pre-adaptation to salt stress and a stronger metabolic reaction (e.g., accumulation of osmolytes) compared to its close relative *Arabidopsis thaliana*, a glycophyte. The protective role of Put in response to UV-B irradiation, in leaves of *T. salsuginea* and other species, was investigated by Radyukina et al. (2017), who concluded that the contribution of bound and free PAs is determined by the species studied and was organ-specific (roots versus leaves).

## Polyamines in Halophytic Crop Species

Most of the species for human consumption belong to glycophytes (salt-sensitive plants), so halophytes can be used as an alternative to traditional agricultural crops, especially in countries with severe climatic conditions (Panta et al., 2014). Quinoa (*Chenopodium quinoa* Willd.) is a halophytic Andean plant, belonging to the Amaranthaceae, with remarkable tolerance to abiotic stress (Bosque-Sánchez et al., 2003; Martínez et al., 2009). This plant has excellent nutritional properties, such as a high protein content and excellent amino acid composition (Repo-Carrasco et al., 2003; Aloisi et al., 2016). Orsini et al. (2011), studying a quinoa accession (BO78) from southern Chile, demonstrated an inverse relationship of Put with tissue levels of Na^+^ or K^+^, thus relating this diamine to the maintenance of a suitable cation/anion balance. A more complete study on four quinoa genotypes, located in coastal-central and southern Chile along a latitudinal gradient, was undertaken by Ruiz-Carrasco et al. (2011), comparing their growth, physiological, and molecular responses, at seedling stage, under saline conditions. With respect to PA levels, all salt-treated quinoa genotypes followed the same pattern, i.e., a sharp decline in Put levels, accompanied by an increase in the ratio of (Spd + Spm) to Put, Spd and Spm being more closely related to vital physiological processes. These researchers concluded that salt-tolerant genotypes might have high levels of both Pro and PAs (Spd/Spm) and could offer a protective function, being useful indicators of salinity adaptation, and potential genotypes for crop improvement. Pottosin and Shabala (2014) and Pottosin et al. (2014) found that PAs were related to the regulation of slow-(SV) and fast-(FV) vacuolar cation channels, as well as vacuolar and (PM) ^+^H pumps and Ca^+2^ pumps of the plasma membrane. Activation of Ca^+2^ efflux by PAs and contrasting effects of PAs on net H^+^ fluxes and membrane potential could contribute to modulate transport processes across the plasma membrane in stress conditions. More recently, Ruiz et al. (2017) studied the PA content and expression of genes involved in PA metabolism in R49 (salares ecotype and northern Chile) and Villarrica (VR, coastal lowlands ecotype, and southern Chile), in response to saline stress. A salt-induced increase was found in the (Spd + Spm): Put ratio, in VR the increase in the ratio occurs later than in R49, although it was significantly higher, distinguishing quinoa genotypes from different habitats. At the gene expression level, it was confirmed that lower Put levels may have resulted from diamine oxidase (*DAO*) up-regulation, arginine decarboxylase (*ADC1*) down-regulation, and (*ADC2*) up-regulation under saline conditions. In both landraces, the higher expression of Spm synthase (*SPMS*) relative to control was consistent with Spm accumulation. In addition, these authors suggested that the PA response found in both landraces could be a consequence of the early up-regulation of 9-*Cis*-epoxycarotenoid dioxygenase (*NCED*), a key enzyme in ABA biosynthesis, which preceded that of the PA biosynthesis genes, and the increase of ABA levels. Pál et al. (2015) have proposed a positive feedback loop in the response to abiotic stress between ABA and PAs.

In recent years, tomato has lost notable growth and yield, especially in arid and semi-arid zones due to intensive irrigation and soil salinization (Munns and Tester, 2008; Gharbi et al., 2017). Comparisons of the glycophyte *Solanum lycopersicum* and its wild relative halophyte *S. chilense* have revealed different ways of tolerating stress, the higher tolerance being related to enhanced levels of salicylic acid (SA), ethylene, and PAs (Gharbi et al., 2016). These researchers have shown an outstanding osmotic adjustment in leaves and greater ethylene production in salt-treated plants of *S. chilense*, correlated with increases, in both shoot and root, of K^+^ concentrations, as occurs in other species such as *Arabidopsis* (Yang et al., 2013). With respect to its biosynthesis, ethylene overproduction proved to be related to 1-aminocyclopropane-1-carboxylic acid synthase (*ACCS2*) gene induction, which has not been reported in *S. lycopersicum*. Jiang et al. (2013) showed that ethylene may contribute to Na^+^/K^+^ homeostasis, under saline stress. Endogenous SA increased in the halophyte in relation to greater stomatal conductance (g_s_), improving the metabolism under stressful conditions. Exogenous SA application and salt may act in synergy on osmolyte synthesis, and a positive linear correlation was recorded between SA content and ethylene synthesis for *S. chilense*. PA levels proved consistently higher in the halophyte than in the glycophyte. Saline stress lowered Put levels and reduced the expression of genes coding for ornithine decarboxylase *ODC*, *ADC1*, and *ADC2* in the halophyte, whereas NaCl increased the expression of those genes in *S. lycopersicum.* Conversely, the tetraamine Spm was only increased in *S. chilense*. Hu et al. (2012) considered high Spd and Spm content to have protective functions in the glycophyte tomato. The application of SA raised PA levels in the aerial part of *S. chilense* (increased *ADC1* and *ADC2* gene expression) so that Put production was partly used for Spd synthesis, but this treatment had no impact in *S. lycopersicum*. However, when the plants were treated with NaCl + SA, the induction of gene expression was not correlated with the accumulation of products, implying a post-transcriptional regulation of PA metabolism. Finally, NaCl slightly induced the expression of the SAM decarboxylase (*SAMDC*) gene in *S. chilense*, while a higher expression was registered in *S. lycopersicum*, suggesting differential responses to salinity in the two species.

## Polyamines as Related to the Potential Use of Halophytes in Phytoremediation

Two of the halophytes most widely studied for their high levels of resistance to salinity stress and drought (Jordan et al., 2002; Martínez et al., 2005) have been the xero-halophytes *Atriplex atacamensis* Phil., a native shrub from Atacama Desert (Chile), and *A. halimus*, a species of Mediterranean areas (Amaranthaceae family). These tolerate salinity by excreting ions (Cl^−^ and Na^+^) through the trichomes located on the surface of their leaves, known as salt bladders, which allow the expulsion of certain amounts of heavy metals (Ben Hassine et al., 2009; Vromman et al., 2011). Given their high biomass production, and capacity to tolerate elevated concentrations of toxic ions (Lutts et al., 2004; Manousaki and Kalogerakis, 2009; Vromman et al., 2011), these could be promising species for phytoremediation. Data reported by Ben Hassine et al. (2009) in *A. halimus*, suggest that free PAs, mainly Spd and Spm, may be involved in the excretion processes and in the regulation of ion fluxes through salt bladders. These researchers suggested that the increase in endogenous PAs (interacting with ATPase or ion channels involved in Na^+^ and Cl^−^ fluxes between epidermis and basal cells of bladders) could reduce Na^+^ influx into mesophyll cells, thereby re-directing Na^+^ flux toward salt bladders. In addition, ABA contributes to PA synthesis and the conversion from bound and conjugated to free soluble forms of PAs, thus favoring salt excretion. Lefèvre et al. (2009, 2010) also found high levels of Put and Spd in a water-stress resistant cell line of *A. halimus*, when tissues were maintained on a medium containing cadmium (Cd), whereas Spm and Dap were higher in the drought stress-sensitive line. PAs are effective in decreasing ionic toxicity, reducing oxidative stress, and restricting the harmful impact of Cd. On the other hand, *A. atacamensis* is able to grow with high external arsenic (As) concentrations in their natural habitat. Vromman et al. (2011), studying the root and leaves of this halophyte, found an increase in free soluble PAs in As-exposed plants. Specifically, Spd and Spm increased at the expense of Put. These researchers hypothesize that these polycationic molecules may assist in arsenate sequestration in the stressed tissues, and therefore should be tested in field trials for the phytomanagement of As-contaminated sites.

Another halophyte common in Mediterranean zones is *Inula chrithmoides*, which belongs to the Asteraceae family. It is a perennial coastal species found on sea cliffs and in salt marshes. Abdel-Wahhab et al. (2008) found medicinal applications by analyzing their metabolite production. This species is found in areas contaminated by Cd, nickel, and salt (Zurayk et al., 2001). In absence of Cd, Ghabriche et al. (2017) have shown that NaCl (100 mM) strongly boosted plant growth and improved the Na^+^: K^+^ ratio in shoot of *I. chrithmoides*. Meanwhile, in Cd-treated plants, NaCl protected this species from ionic toxicity and helped reduce Cd absorption and translocation. PAs increased in response to both NaCl and CdCl_2_ (mainly the bound PA fraction) in leaves and roots, and this increase could be related to protective functions of PAs on endogenous cellular structures. In rice, Li et al. (2013) found that Cd decreased the activities of glutamine synthetase and glutamate synthase, enzymes of N assimilation pathway, and therefore increased NH_4_^+^ and arginine content (a precursor of Put). Thus, Ghabriche et al. (2017) proposed that the Cd-induced alteration of the N cycle was the main factor responsible for altering PA levels in *I. chrithmoides*.

The halophyte *Kosteletzkya pentacarpos* (formerly *K. virginica*) is a perennial wetland species (Malvaceae family) and a promising plant for saline agriculture (He et al., 2003). This plant grows in coastal areas contaminated by industrial activity (Lutts and Lefèvre, 2015). Zhou et al. (2018a,b) found that salt and heavy metals altered the endogenous PA content, providing protection under these stress conditions. Heavy metals augmented ethylene synthesis, but NaCl depressed it in plants exposed to Cd or the combined treatment (Cd + Zn) but not with Zn. PA concentrations were altered by Cd (Spd and Spm decreased concomitantly with Put accumulation). These researchers concluded that PA profile could be the result of ethylene overproduction in Cd-treated plants because SAM is a precursor of ethylene, Spd, and Spm. Inhibition of ethylene synthesis increased Spd and Spm level and reduced Cd accumulation.

## Polyamines in Euhalophytes

In *Cymodocea nodosa*, a seagrass, PAs are distributed in all plant tissues, but mainly the apical section of the rhizome (Marián et al., 2000). Zarranz Elso et al. (2012) showed a decline in PAs during embryo development (free PAs), and an increase during germination, indicating the key role of these growth regulators in the first steps of the zygotic and germination stages. Exogenous Spd helps improve *Fv/Fm*-values, exerting a protective role in maintaining the photosynthetic apparatus. Ioannidis and Kotzabasis (2007), Hamdani et al. (2011), and Malliarakis et al. (2015) found that application of PAs (Spd and Spm) could restore PSII efficiency, via electrostatic interactions with extrinsic and intrinsic, and PSII thylakoid proteins, thereby stabilizing them. These results indicate that *C. nodosa* requires elevated PA levels to survive in high salinity. *Prosopis strombulifera*, a common spiny shrub, belonging to the family Fabaceae (subfamily Mimosoideae) is distributed in arid regions (of North and South America). Reginato (2009) and Reginato et al. (2012) considered this plant to be a true “euhalophyte” for NaCl, being less tolerant to Na_2_SO_4_, although a partial improvement was found with two salt mixtures. Put accumulation exerted a beneficial effect in NaCl-treated plants, stimulating growth and the antioxidative defense system. However, low Put levels, in leaves, were correlated with inhibition of shoot growth, associated with Na_2_SO_4_ toxicity. Cad and Dap were related with stress symptoms, and possibly, the activities of PA oxidases.

## Author Contributions

MB and M-PC revised the Bibliography. MB wrote the manuscript.

## Conflict of Interest Statement

The authors declare that the research was conducted in the absence of any commercial or financial relationships that could be construed as a potential conflict of interest.
